# Reproducibility of Blood Lactate Concentration Rate under Isokinetic Force Loads

**DOI:** 10.3390/sports6040150

**Published:** 2018-11-20

**Authors:** Nico Nitzsche, Lutz Baumgärtel, Christian Maiwald, Henry Schulz

**Affiliations:** 1 Technische Universität Chemnitz, Center of Sport and Health Promotion, 09126 Chemnitz, Germany; 2 Technische Universität Chemnitz, Institute of Human Movement Science and Health, 09126 Chemnitz, Germany; lutz.baumgaertel@hsw.tu-chemnitz.de (L.B.); christian.maiwald@hsw.tu-chemnitz.de (C.M.); henry.schulz@hsw.tu-chemnitz.de (H.S.)

**Keywords:** anaerobic energy, lactate, resistance exercise, alactic time interval, energy metabolism

## Abstract

(1) Background: Maximum isokinetic force loads show strongly increased post-load lactate concentrations and an increase in the maximum blood lactate concentration rate ($\dot{V}$La_max_), depending on load duration. The reproducibility of $\dot{V}$La_max_ must be known to be able to better assess training-related adjustments of anaerobic performance using isokinetic force tests. (2) Methods: 32 subjects were assigned to two groups and completed two unilateral isokinetic force tests (210° s^−1^, Range of Motion 90°) within seven days. Group 1 (n = 16; age 24.0 ± 2.8 years, BMI 23.5 ± 2.6 kg m^−2^, training duration: 4.5 ± 2.4 h week^−1^) completed eight repetitions and group 2 (n = 16; age 23.7 ± 1.9 years, BMI 24.6 ± 2.4 kg m^−2^, training duration: 5.5 ± 2.1 h week^−1^) completed 16 repetitions. To determine $\dot{V}$La_max_, capillary blood (20 µL) was taken before and immediately after loading, and up to the 9th minute post-load. Reproducibility and variability was determined using Pearson and Spearman correlation analyses, and variability were determined using within-subject standard deviation (S_w_) and Limits of Agreement (LoA) using Bland Altman plots. (3) Results: The correlation of $\dot{V}$La_max_ in group 1 was r = 0.721, and in group 2 r = 0.677. The S_w_ of $\dot{V}$La_max_ was 0.04 mmol L^−1^ s^−1^ in both groups. In group 1, $\dot{V}$La_max_ showed a systematic bias due to measurement repetition of 0.02 mmol L^−1^ s^−1^ in an interval (LoA) of ±0.11 mmol L^−1^ s^−1^. In group 2, a systematic bias of −0.008 mmol L^−1^ s^−1^ at an interval (LoA) of ±0.11 mmol L^−1^ s^−1^ was observed for repeated measurements of $\dot{V}$La_max_. (4) Conclusions: Based on the existing variability, a reliable calculation of $\dot{V}$La_max_ seems to be possible with both short and longer isokinetic force loads. Changes in $\dot{V}$La_max_ above 0.11 mmol L^−1^ s^−1^ due to training can be described as a non-random increase or decrease in $\dot{V}$La_max_.

## 1. Introduction

Maximum anaerobic loads result in increased lactate accumulation and, depending on the load duration (10 s vs. 30 s), in higher concentrations of post-load lactate [1]. Anaerobic performance can be determined using the blood lactate concentration. The anaerobic performance expressed by lactate concentration rate represents the stress of glycolysis [2]. The change in lactate concentration is determined over load duration. To avoid underestimating the rate of lactic acid formation, it is necessary to consider the alactacid time interval of the load duration. Up to now, the efficiency of anaerobic energy supply has been primarily investigated using running and bicycle ergometer loads [1,3,4,5,6]. This has already shown good to very good reproducibility of maximum power (P_max_) [4,5]. Adam et al. [5] found very good reproducibility of $\dot{V}$La_max_ in anaerobic bicycle sprints over 15 s. When the load duration increases, Heck and Schulz [3] assume a reduction of $\dot{V}$La_max_. Empirical evidence of this was provided by Hauser [7].

Isokinetic force tests to determine muscle performance are important in sports practice and research [8]. Performing isokinetic force tests at maximum performance may show the performance of anaerobic metabolism. The strong metabolic stress of the anaerobic energy metabolism at maximum isokinetic loads is characterized by strongly increased post-load lactate concentrations and a resulting maximum lactate concentration rate ($\dot{V}$La_max_) [9]. $\dot{V}$La_max_ increases with accelerations in movement velocity under isokinetic force loads [10]. Studies on anaerobic performance under isokinetic force loads have shown good to excellent reproducibility of performance and torque [11,12,13,14,15]. Furthermore, Bosquet et al. [16] showed a high correlation of the “total work” between bicycle sprints (Wingate-Test) and isokinetic leg extension movements. Similar results were obtained when comparing a Wingate-Test and a sprint test in running [6,17].

Compared to an isokinetic bicycle sprint, it is possible to make statements about the local performance of muscles using an isokinetic strength test. This can be important for sports with high strength components and dominant anaerobic demands on energy metabolism, such as martial arts, gymnastics, and strength sports, such as CrossFit or weight lifting.

However, due to the lower active muscle content in the isokinetic force test compared to the bicycle sprint and therefore changed lactate invasion and elimination, the absolute glycolysis rates are not directly comparable [9]. A unilateral isokinetic strength test with one leg primarily stresses the leg muscles. A bicycle test also involves parts of the trunk, in addition to both legs. It is therefore questionable whether the results of high reproducibility for $\dot{V}$La_max_ in the bicycle sprint can be transferred to isokinetic force tests. So far, no investigations on the reproducibility of $\dot{V}$La_max_ under isokinetic force loads have been carried out. Knowledge of the reproducibility and thus the variability of $\dot{V}$La_max_ in isokinetic force tests is important to explain the changes in local anaerobic performance. This makes it easier to interpret the effects of training intervention (especially local resistance training) on anaerobic performance. Since $\dot{V}$La_max_ appears to depend on the duration of an anaerobic test, it should be clarified how variability presents itself at different load durations. The aim of the present study was to investigate the reproducibility of $\dot{V}$La_max_ under isokinetic force loads as a function of load duration (t_load_).

## 2. Materials and Methods

Thirty two trained male subjects (age 23.9 ± 2.4 years, body height 177.0 ± 8.3 cm, body mass 76.3 ± 11.5 kg, BMI 24.1 ± 0.1 kg m^−2^, practiced sports: soccer, endurance, martial arts, strength training) agreed to voluntary participation in the study after receiving oral and written information. The study was approved by the local ethical committee (V-297-17-HS-Lactate-18102018) and conducted in accordance with the Declaration of Helsinki. The subjects were then assigned to two groups and completed two single-legged isokinetic force tests (CON-TREX^®^ MJ, PHYSIOMED^®^, Schnaittach, Germany) with unilateral knee flexion and extension movements (Range of Motion 90°) within seven days of each other. The upper body was immobilized with belts and the non-active leg was also immobilized. The subjects were instructed to perform each movement (flexion and extension) at maximum power. The movement velocity was chosen as 210° s^−1^, since this was the highest $\dot{V}$La_max_ was found in pretests.

Group 1 (age 24.0 ± 2.8 years, body height 174.8 ± 10.2 cm, body mass 72.3 ± 12.1 kg, BMI 23.5 ± 2.6 kg m^−2^, training scope per week 4.5 ± 2.4 h) completed eight repetitions, and group 2 (age 23.7 ± 1.9 years, body height 178.8 ± 5.2 cm, body mass 78.9 ± 8.5 kg, BMI 24.6 ± 2.4 kg m^−2^, training scope per week 5.5 ± 2.1 h) completed 16 repetitions.

**Figure 1:**
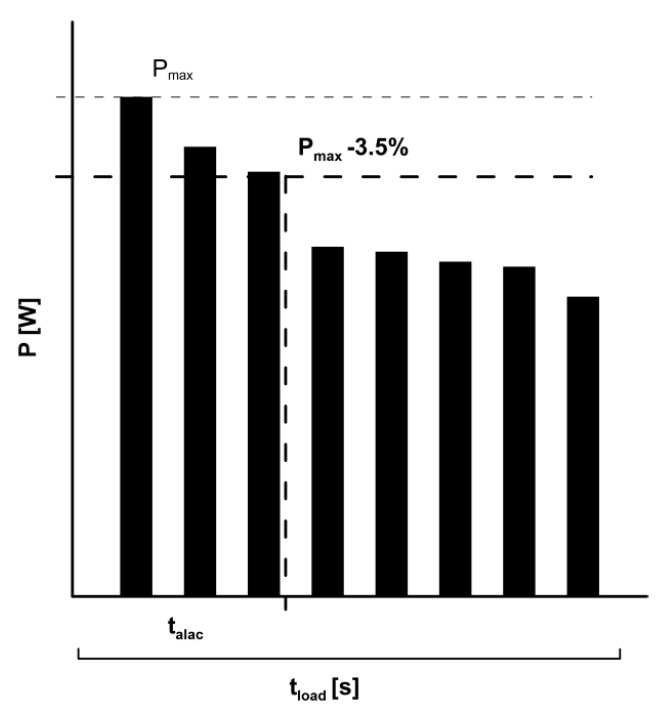
Schematic representation of determining the alactacide time interval (t_alac_) on the basis of power (P).

Before each measurement, two capillary blood samples were taken from the earlobe to determine resting lactate (RL). Immediately after loading, capillary blood (20 µL) was drawn every 30 s until the third minute post-loading. Subsequently, capillary blood was taken every minute until the 9th minute post-loading. The calculation (1) of $\dot{V}$La_max_ was done according to the model by Mader [2] and Heck and Schulz [3]. The maximum blood lactate in the post-loading period (La_max_), resting lactate (RL), test duration (t_load_), and the alactic time interval (t_alac_) were used to determine $\dot{V}$La_max_ [3]. The alactic time interval (Figure 1) is the range from the start of the test to the time at which the maximum power (P_max_) has dropped by 3.5% [5,18].

(1) $$\dot{V}{\mathrm{La}}_{\max}=({\mathrm{La}}_{\max}-\mathrm{RL})*(t_{\mathrm{load}}-t_{\mathrm{alac}}){}^{-1},$$

The Shapiro-Wilk test was used to check the normal distribution of the variables $\dot{V}$La_max_, La_max_, t_load_, RL, P_max_ and t_alac_. A paired t-test was performed to test for significant differences in RL and t_load_ between Test 1 and Test 2. For normally distributed data, linear correlations were tested using Pearson’s correlation. The Spearman’s rho was used for data that showed no normal distribution. Cohen and Manion [19] was used to assess the linear relationship between Test 1 and Test 2. Bland and Altman plots (BA plots) were used to test the dependence of the measurement differences between Test 1 and Test 2 for homoscedasticity [20]. The Limits of Agreement (LoA) were calculated from the mean difference (MW_Diff_) ± standard deviation * 1.96. Non-normally distributed differences for t_alac_ and P_max_ were calculated using the non-parametric LoA method (median_Diff_) based on the 2.5 (Q_0.025_) and 97.5 percentiles (Q_0.975_) [21]. The measurement error as within-subject standard deviation (S_w_) was calculated to determine the variability of the variables La_max_, $\dot{V}$La_max_, P_max_, and t_alac_ [22]. The S_w_ (S_w-norm_ in %) was normalized by dividing the S_w_ by the group mean * 100. The significance level was 5%.

## 3. Results

### 3.1. T-Tests and Correlations

In group 1, an RL of 0.86 ± 0.22 mmol L^−1^ was present before Test 1 and 0.91 ± 0.35 mmol L^−1^ in Test 2 (*p* > 0.05). Group 2 showed an RL of 0.83 ± 0.41 mmol L^−1^ before Test 1 and an RL of 0.76 ± 0.18 mmol L^−1^ (*p* > 0.05) before Test 2. In both groups, t_load_ did not differ significantly between the two tests (*p* > 0.05).

In both groups, high correlations between the tests were determined for mean maximum torque, mean maximum power, and maximum power (Table 1). The physiological quantities La_max_ and $\dot{V}$La_max_ of group 1 showed a correlation between both tests of r = 0.688 and r = 0.721. In group 2, there was a correlation of r = 0.821 (La_max_) and r = 0.677 ($\dot{V}$La_max_). These can be considered high linear correlations. With regard to t_alac_, a high correlation of r > 0.665 was observed in group 1 between test 1 and test 2. Group 2 showed a mean linear correlation of r = 0.482 between test 1 and test 2.

### 3.2. Variability of Measurement

**Figure 2:**
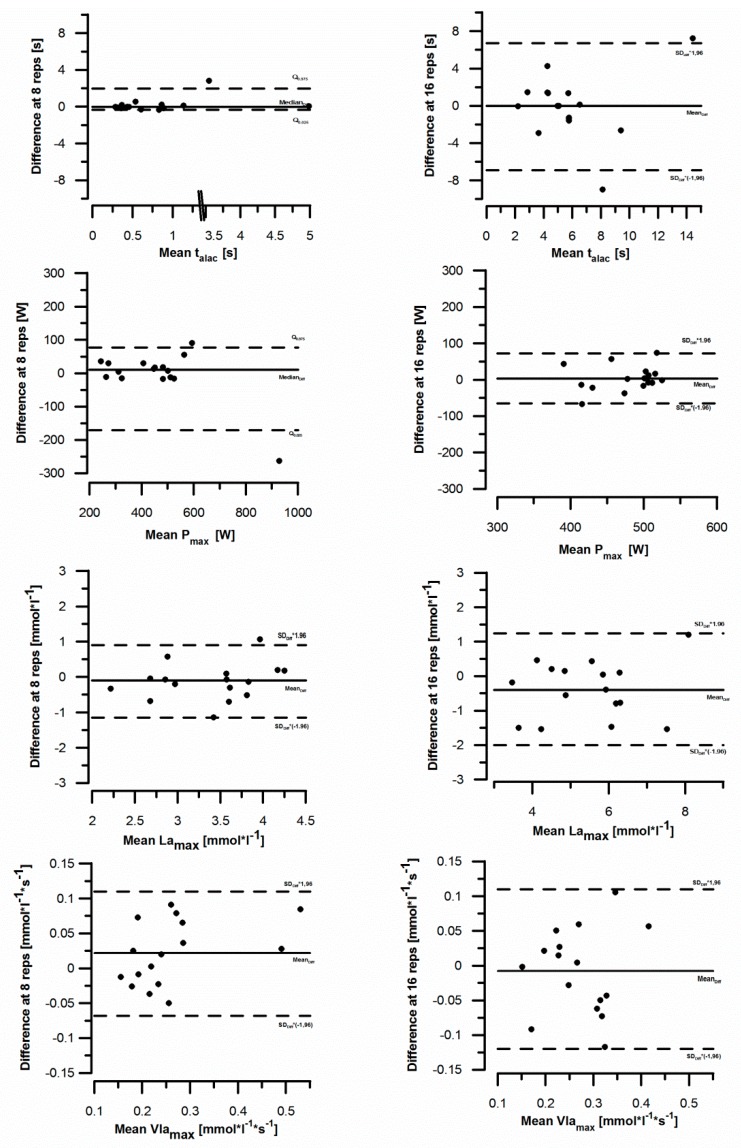
Bland Altman plots for parameters t_alac_, P_max_, La_max_, $\dot{V}$La_max_. The differences between the two measurements are plotted against the mean value.

The S_w_ variability for La_max_, P_max_, and $\dot{V}$La_max_ ranged between 5% and 16% (Table 2). t_alac_ showed S_w_ between 40% and 50%. The variability of $\dot{V}$La_max_ in both groups was 0.04 mmol L^−1^ s^−1^ (S_w-norm_: 13.47% to 15.78%). Figure 2 shows the systematic bias $\dot{V}$La_max_ quantities using BA plots for both groups.

For group 1, the BA plots for La_max_ showed a systematic bias on the repeatability (MW_Diff_) of −0.12 mmol L^−1^ at an interval (LoA) of ±0.89 mmol L^−1^. At P_max_ the median_Diff_ was 10.7 watts at a lower LoA (Q_0.025_) of - 170.98 watts and an upper LoA (Q_0.975_) of 77.35 watts. t_alac_ showed a median_Diff_ of −0.02 s with a lower LoA (Q_0.025_) of −0.33 s and an upper LoA (Q_0.975_) of 1.96 s. At $\dot{V}$La_max_, a systematic bias of 0.02 mmol L^−1^ s^−1^ could be determined by repeating the measurement at an interval (LoA) of ±0.11 mmol L^−1^ s^−1^.

For group 2, a systematic bias on the measurement repetition (MW_Diff_) was −0.38 mmol L^−1^ with an interval (LoA) of ± 1.24 mmol L^−1^ was found based on the BA plots for La_max_. The MW_Diff_ of P_max_ was determined as 3.68 watts at an LoA of ± 72.1 watts. The t_alac_ showed an MW_Diff_ of 0.11 s with an LoA of ± 6.70 s. The systematic bias on measurement repetition for $\dot{V}$La_max_ was −0.008 mmol L^−1^ s^−1^ at an interval (LoA) of ±0.11 mmol L^−1^ s^−1^.

The Bland and Altman plots did not show a tendency in the measurement differences with respect to the mean value of the variables for either group. Thus homoscedasticity of the measurement differences was assumed.

## 4. Discussion

The aim of the study was to investigate the reproducibility of $\dot{V}$La_max_ under isokinetic force loads. For this purpose, single-legged maximum leg flexion and extension movements with different load durations were performed on two groups of test subjects using a test and retest design. With respect to the target variable $\dot{V}$La_max_, the results of the linear correlation showed a high correlation in the test with eight repetitions and a moderate correlation in the test with 16 repetitions. The variability of the S_w_ can be considered as low for the investigated physiological values. Adam et al. [5] found similar results for maximum bicycle sprints. 

$\dot{V}$La_max_ is estimated on the basis of values, which are, in part strongly dependent on coordinative, motivational, and metabolic factors. This physiological size can potentially be affected by numerous determinants. Maximum lactate and duration of the t_alac_ is crucial with comparable load time and preload lactate concentration. In this context, t_alac_ showed the highest variability compared to the other values determining $\dot{V}$La_max_. The data suggest that the variability between subjects is similar to that between repeated measurements.

In terms of t_alac_, it would be difficult to distinguish between intraindividual changes and interindividual characteristics. t_alac_ is the time interval at which the maximum power has been reduced by 3.5%. If the power maximum occurs too late or the power drop can be delayed, t_alac_ increases and consequently $\dot{V}$La_max_ increases. For approx. 10 s of running load, Heck and Schulz [3] assume a t_alac_ of approx. four seconds, which increases with increasing running distance, and thus depends on the running strategy. At maximum isokinetic force loads, Nitzsche et al. [9] calculated a t_alac_ of approx. three seconds. From a physiological point of view, t_alac_ represents the time interval which is secured by “high-energy substrates” creatine phosphate (PCr) and adenosine triphosphate (ATP) to generate or maintain a maximum performance. At loads that provide maximum performance over a short time, PCr and ATP (5 to 3.5 mmol L^−1^) drop rapidly, which leads to a drop in performance due to the lower flow rate of phosphates via anaerobic glycolysis [3,23]. Therefore, P_max_ cannot physiologically occur if t_alac_ is delayed. The present study showed lower t_alac_ at eight repetitions and higher t_alac_ at 16 repetitions. Between Test 1 and Test 2, group 1 (8 reps) had a higher linear correlation than group 2 (16 reps). This suggests that the subjects in group 2 (16 reps) may not have achieved maximum performance at the beginning of the test, but may have delayed it. Therefore, $\dot{V}$La_max_ tends to be overestimated.

Since all test subjects were familiar with the test through preliminary examinations, coordinative reasons as well as learning and training effects can be excluded. Wittekind et al. [24] were able to determine “pacing” strategies for “all out” loads. They observed reduced performance at the beginning of longer loading periods (45 s) compared to shorter loading periods (10 s). It is possible that a strategy has been used here, consciously or unconsciously, with the aim of achieving high performance with low performance loss over the entire test period. This influencing factor for determining $\dot{V}$La_max_ should be investigated in the future using a blinded design. Adam et al. [5] found differences in t_alac_ of up to one second on average during three repetitions of an isokinetic bicycle sprint. In the present study, the differences averaged less than 0.5 s. Introducing a time constant to minimize the variability of $\dot{V}$La_max_ would be conceivable for t_alac_. Determining $\dot{V}$La_max_ would thus be less strongly influenced by a distorted t_alac_. 

Methodologically, it should be noted that direct comparisons of the variability of $\dot{V}$La_max_ between the two loading periods are difficult to assess, as they were two independent groups of test persons. The allocation was randomized, but was not yet balanced due to the small sample size. A dependent design would make the comparison and its conclusions more reliable. In particular, the two groups of test persons showed different performances despite comparable training frequencies. With regard to performance, there was a significantly greater variability between the subjects in group 1, possibly having an influence on the higher correlations of $\dot{V}$La_max_ compared to group 2. The variability of La_max_ may be dependent on carbohydrate intake and the level of the glycogen stores [25,26]. Since no dietary protocol was kept in the present study, there is uncertainty about possible influences of the test persons’ diet.

## 5. Conclusions

A reliable reproducibility of $\dot{V}$La_max_ at isokinetic force loads is given both during short (8 reps.) and longer loads (16 reps.). To minimize any distortions of t_alac_ by possible strategies of power output, using shorter load durations is preferable with regard to practical application. Furthermore, a constant for t_alac_ may control the influence of the power output. Changes in the $\dot{V}$La_max_ of over 0.11 mmol L^−1^ s^−1^ due to training can be described as a non-random increase or decrease in the $\dot{V}$La_max_.

## Figures and Tables

**Table 1:** Test-Retest results (mean and standard deviation) of both groups and the correlation coefficient r (*p*-value).

Groups	Group 1 with 8 reps			Group 2 with 16 reps		
Parameter	Test 1	Test 2	r	Test 1	Test 2	r
RL (mmol L^−1^)	0.86 ± 0.22	0.91 ± 0.35		0.83 ± 0.41	0.76 ± 0.18	
t_load_(s)	12.1 ± 1.02	11.5 ± 1.03		23.1 ± 0.31	23.0 ± 0.28	
M_max-mean_ (Nm)	143.8 ± 43.3	145.5 ± 45.1	0.983 (0.000)	143.8 ± 16.9	142.6 ± 18.9	0.840 (0.000)
P_max-mean_ (Watt)	424.6 ± 182.2	424.9 ± 142.6	0.946 (0.000)	419.6 ± 40.2	419.9 ± 40.1	0.753 (0.001)
P_max_ (Watt)	457.9 ± 193.5	455.9 ± 146.9	0.938 (0.000)	476.0 ± 42.8	479.7 ± 49.9	0.726 (0.001)
La_max_ (mmol L^−1^)	3.44 ± 0.59	3.31 ± 0.70	0.688 (0.002)	5.66 ± 1.33	5.27 ± 1.44	0.821 (0.000)
$\dot{V}$La_max_ (mmol L^−1^ s^−1^)	0.25 ± 0.11	0.27 ± 0.11	0.721 (0.002)	0.27 ± 0.07	0.26 ± 0.07	0.677 (0.004)
t_alac_ (s)	0.97 ± 1.1	1.1 ± 1.5	0.665 (0.005)	5.87 ± 3.1	5.76 ± 3.6	0.482 (0.059)

**Table 2:** Within-subject standard deviation S_w_ and (S_w-norm_) of both groups.

Parameter	Group 1	Group 2
P_max_ (Watt)	51.75 (11.33%)	24.05 (5.03%)
La_max_ (mmol L^−1^)	0.37 (10.92%)	0.63 (11.54%)
$\dot{V}$La_max_ (mmol L^−1^ s^−1^)	0.04 (13.47%)	0.04 (15.78%)
t_alac_ (s)	0.52 (49.28%)	2.38 (40.9%)
